# An Achilles' heel for methicillin-resistant *Staphylococcus aureus*? Re-evaluating β-lactam susceptibility of MRSA

**DOI:** 10.1128/aac.00374-26

**Published:** 2026-07-06

**Authors:** Neta Petersiel, Benjamin P. Howden, Stefano Giulieri, Steven Y. C. Tong

**Affiliations:** 1 Department of Infectious Diseases, The University of Melbourne, The Peter Doherty Institute for Infection and Immunity 534133, Melbourne, Victoria, Australia; 2 Department of Microbiology and Immunology, The University of Melbourne, The Peter Doherty Institute for Infection and Immunity 534133, Melbourne, Victoria, Australia; 3 Microbiological Diagnostic Unit Public Health Laboratory, Department of Microbiology and Immunology, University of Melbourne, The Peter Doherty Institute for Infection and Immunity 534133, Melbourne, Victoria, Australia; 4 Centre for Pathogen Genomics, The University of Melbourne, Melbourne, Victoria, Australia; 5 Department of Infectious Diseases, Austin Health 740438, Heidelberg, Victoria, Australia; 6 Victorian Infectious Diseases Service, The Royal Melbourne Hospital, The Peter Doherty Institute for Infection and Immunity 534133, Melbourne, Victoria, Australia; 7 Methods and Implementation Support for Clinical and Health Research Hub, School of Population and Global Health, Melbourne Medical School, University of Melbourne, Melbourne, Australia; Samsung Medical Center, Seoul, Republic of Korea

**Keywords:** MRSA, β-lactams, phenotypes

## Abstract

Methicillin-resistant *Staphylococcus aureus* (MRSA) infections (i.e., carrying the *mec* gene) are challenging to treat due to their inherent resistance to most antistaphylococcal β-lactams. Given the poor outcome of severe MRSA infections and limitations of the current approved treatments, there is growing interest in repurposing β-lactams as part of a treatment strategy for these infections. Here, we review the current literature on β-lactam susceptibility phenotypes in MRSA. We describe four phenotypes: oxacillin-susceptible, sensitivity to combinations of β-lactam-β-lactamase inhibitors, NaHCO_3_ responsiveness, and synergy between classes of β-lactams that target different penicillin-binding proteins. For each phenotype, we discuss the genetic background, known explanatory biological mechanisms, and associations between MRSA β-lactam susceptibility phenotype and treatment response to β-lactam therapy.

## INTRODUCTION

*Staphylococcus aureus* bloodstream infections (BSIs) are life-threatening and carry high morbidity and a 30-day mortality of 18–21% (1). Methicillin-resistant *S. aureus* (MRSA) bloodstream infections are associated with a higher crude mortality rate than methicillin-susceptible *S. aureus* (MSSA) BSI (2), in part due to host factors, limited treatment options, and shortcomings of vancomycin (3, 4). Alternative and adjunctive treatment approaches include consideration of using anti-staphylococcal β-lactams. Indeed, there is an underappreciation that MRSA strains can have a wide variation in susceptibility to β-lactams.

## β-LACTAM RESISTANCE IN MRSA

**Fig 1:**
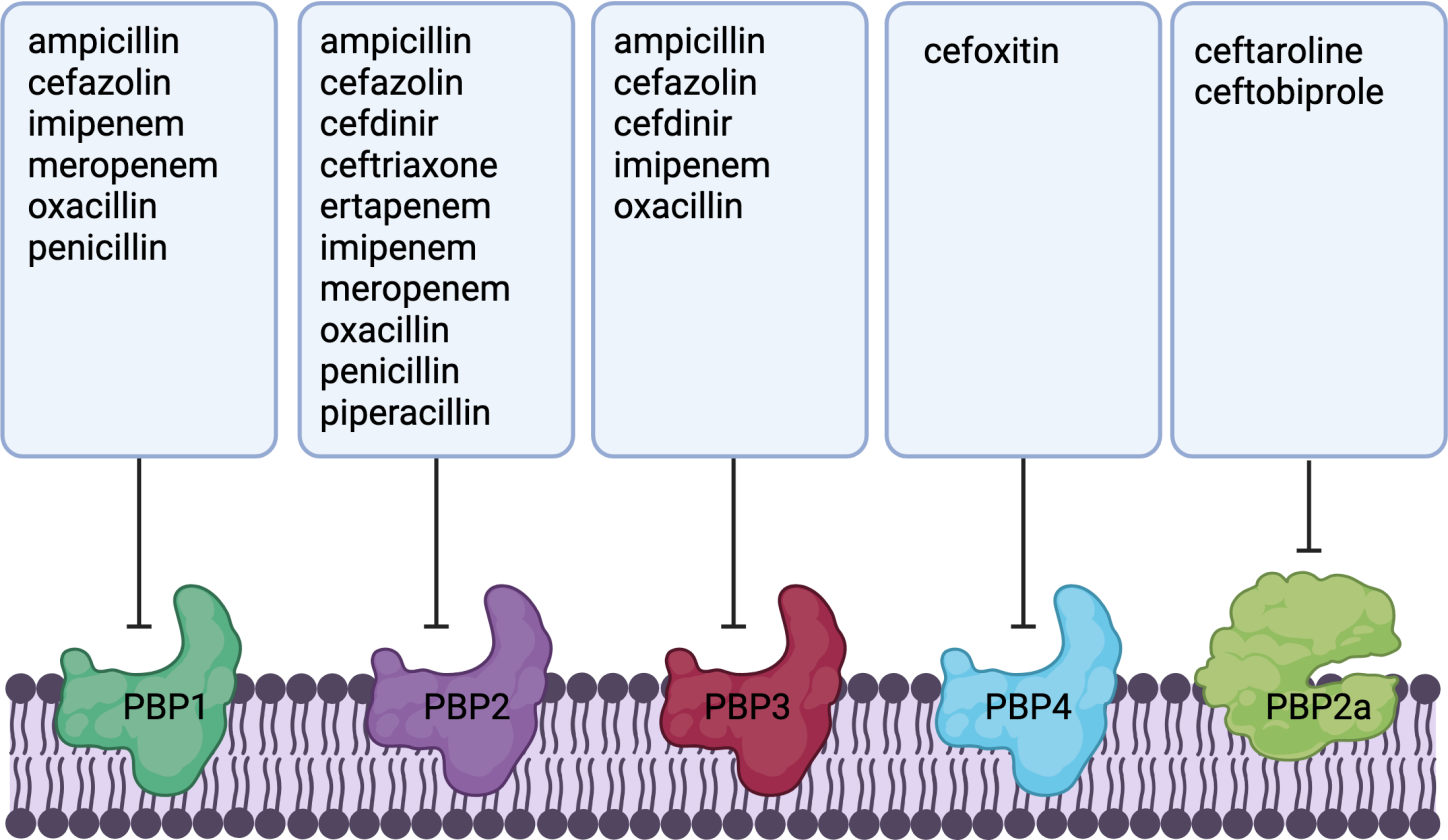
*Staphylococcus aureus* penicillin-binding proteins (PBPs) and selected β-lactams that preferentially inhibit them (7, 10, 13–19).

β-Lactams act by targeting the transpeptidase domain of penicillin-binding proteins (PBPs) and thereby inhibiting cell wall synthesis (5). *S. aureus* carries four native PBPs, PBP1-4, all of which participate in cross-linking of peptidoglycan through their transpeptidase domain. The affinity of β-lactams to the transpeptidase of these PBPs differs from agent to agent. While some β-lactams are non-selective and can bind to different PBPs with similar affinity, others bind selectively to a specific PBP (6–12) (Fig. 1).

In the overwhelming majority of cases, anti-staphylococcal β-lactams are considered ineffective against MRSA due to the presence of a fifth accessory PBP, PBP2a. The active site of PBP2a is hidden in a cleft, preventing most β-lactams from binding, meaning that MRSA can continue to form peptidoglycan in the presence of most β-lactams. The active site can become accessible by activation of an allosteric domain, leading to a conformational change in PBP2a (20). The anti-MRSA cephalosporins, ceftaroline (13) and ceftobiprole (14), are unique in their high affinity for binding to PBP2a, and have been clinically approved (21, 22) to treat MRSA infections. However, access to ceftaroline and ceftobiprole is limited by availability and cost in most regions of the world, and reports of resistance (mainly due to *mec* and *pbp4* mutations) are emerging (23). Here, we discuss susceptibility of MRSA to β-lactams outside of ceftaroline and ceftobiprole.

PBP2a is typically encoded by the *mecA* gene, which is part of the *mec* gene complex along with the regulatory genes *mecI* and *mecR*. While other *mec* genes have been reported, such as *mecB* (24) and *mecC* (25), *mecA* remains by far the most common in clinical MRSA strains. The expression of *mecA* is regulated by repressors, *mecI* or *blaI,* and inducers, *mecR* or *blaR (26, 27*). In response to the presence of β-lactams, *mecR/blaR* cleaves *mecI/blaI,* resulting in increased expression of *mecA*. The *mec* gene complex is located on the SCC*mec* cassette (28, 29), which can be classified into types based on a combination of three elements: the *mec* complex, the recombinase gene (*ccr*), and the joining region (J region) (30). There are currently 15 recognized types of SCC*mec* (I–XV) (31), the most common being types I, II, and III that are carried by MRSA lineages that were typically classified as healthcare-associated (HA-MRSA), and types IV and V that are carried by MRSA lineages that were usually regarded as community-associated (CA-MRSA). The distinction between HA- and CA-MRSA is somewhat arbitrary (32), and as more genomic data become available, it has become clear that certain clones typically associated with healthcare infections (like ST22) have SCC*mec* types associated with CA-MRSA.

**Fig 2:**
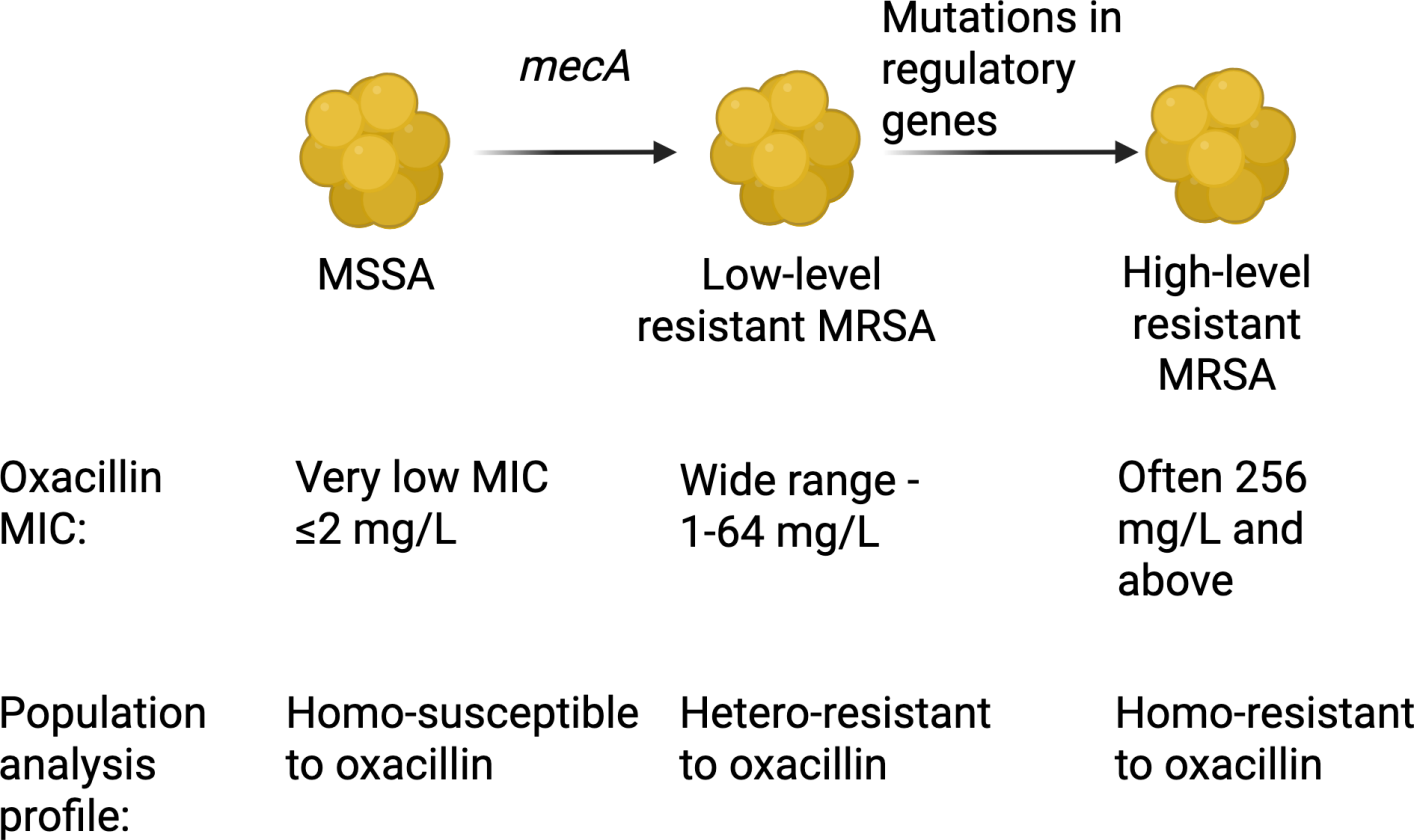
Stepwise acquisition of β-lactam resistance in *Staphylococcus aureus*. After acquisition of *mecA*, *S. aureus* expresses a low-level resistance to β-lactams (low MIC and a hetero-resistant phenotype on population analysis profile [PAP]). After acquiring mutations in regulatory genes, *S. aureus* expresses a high level of β-lactam resistance (high MIC and homo-resistant phenotype on PAP). MIC, minimal inhibitory concentration.

Although *mecA* is essential for expression of methicillin resistance in *mec*-positive MRSA, its presence alone typically confers only low-level β-lactam resistance, as demonstrated in studies where *mecA* is introduced into MSSA (33, 34) (Fig. 2). The low-level resistance in these lab-derived strains is due to a heteroresistant phenotype, in which the vast majority of cells (>99%) are phenotypically susceptible, while a small subpopulation exhibits high-level resistance (35). The hetero-resistant phenotype can be revealed by a technique called population analysis profiling (PAP), where the number of colony-forming units of an isolate is plotted against serial dilutions of antibiotic concentrations (36). The frequency of the resistant subpopulation varies by strain and is reproducible (29, 35). *In vitro*, under antibiotic exposure, the heteroresistant phenotype can revert to a homoresistant phenotype where the entire population expresses high minimum inhibitory concentration (MIC) to β-lactams (35). High-level resistance to β-lactams comes at a fitness cost to MRSA, reflected by slow growth rates of highly resistant strains (37). In contrast, mutations in *mecA* associated with reduced resistance to β-lactams have been shown to confer a fitness benefit (38). PAP is labor-intensive and is not used in clinical labs, where MICs are used to assess the level of β-lactam resistance. Broadly, the MIC levels can be used as a surrogate for heteroresistance vs homoresistance (35). As recently reviewed by Bilyk et al. (39), high-level oxacillin-resistant (OR)-MRSA derivatives of low-level β-lactam resistant strains carry mutations in genes that have been implicated in the (p)ppGpp-mediated stringent response, such as *relA* (40–43) and *gdpP (*33), or in transcription such as *RpoB/C* (33, 41–44).

In a minority of cases, β-lactam resistance can be mutational and *mec* independent (45, 46). These isolates lack a *mec* gene and typically carry mutations in *gdpP* or *pbp4* and their promoter. They were previously known as BORSA (borderline oxacillin-resistant *S. aureus*) (47) and thought to be linked to β-lactamase hyperproduction; however, more recently, the terms MIONSA (mec-independent oxacillin non-susceptible *S. aureus*) or MLRM (methicillin-resistant lacking mec) are used. In this review, we will not further discuss *mecA*-negative strains and will now address β-lactam susceptibility phenotypes in *mec*-positive MRSA.

## COULD β-LACTAMS HAVE A ROLE IN THE TREATMENT OF MRSA?

**Fig 3:**
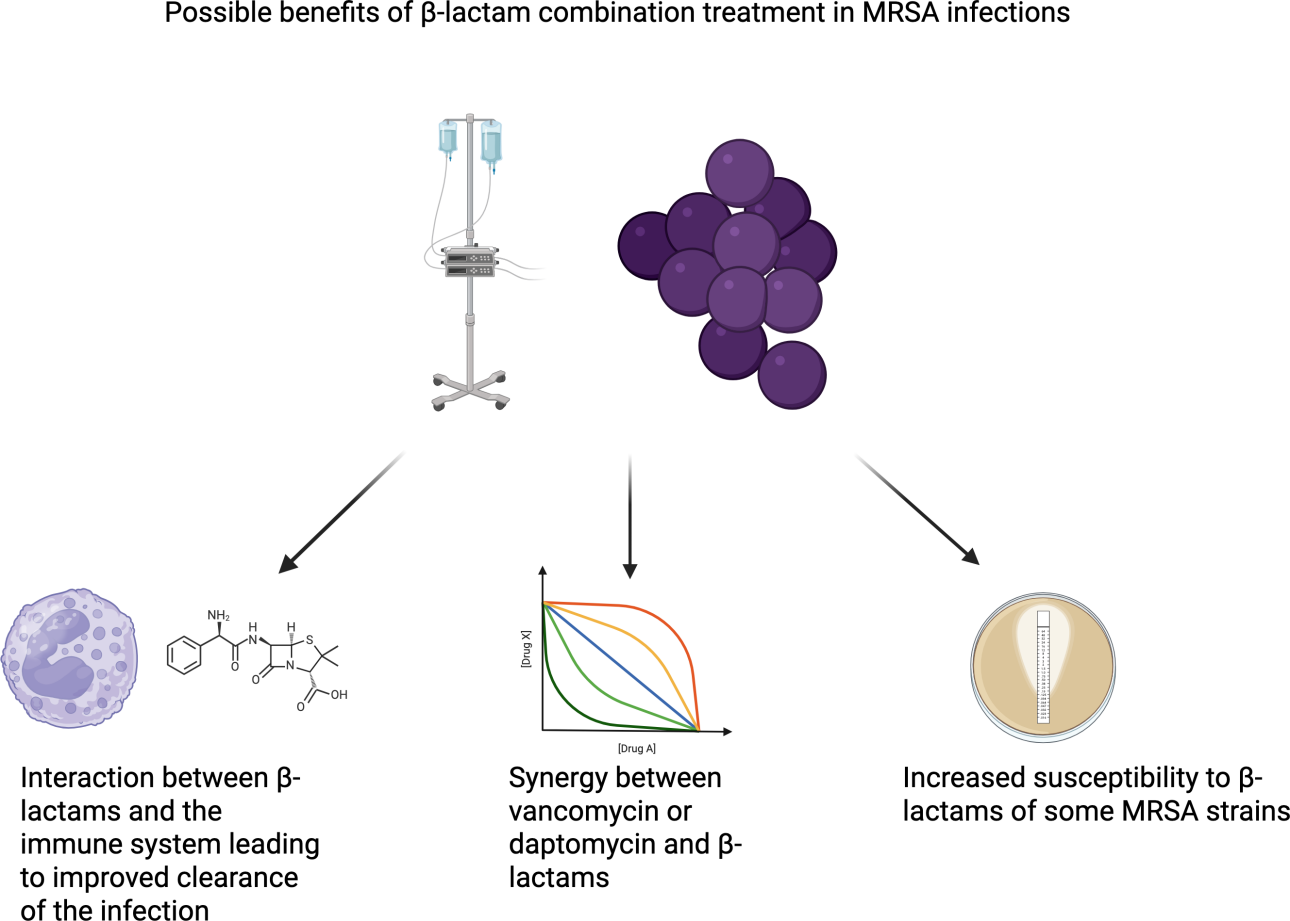
Possible benefits of β-lactam combination treatment in methicillin-resistant *Staphylococcus aureus* (MRSA) infections.

Current guidelines recommend vancomycin or daptomycin for severe MRSA infections (48). However, an increasing body of data suggests that β-lactams could have a role in the treatment of MRSA infections. In recent years, *in vitro* studies have reported that some MRSA strains are phenotypically more susceptible to β-lactams than others (38, 49, 50). *In vivo* studies (38, 51, 52) using various animal models have shown that *in vitro* β-lactam susceptibility can predict infection clearance from tissues and blood. Moreover, there are limitations to the currently recommended methods for measuring MICs of β-lactams to predict clinical outcomes in staphylococcal infections. As the *in vitro* MICs may not accurately represent *in vivo* MICs in tissues during an infection (53), authors such as Sakoulas and Nizet (54) highlight that the media used for antimicrobial susceptibility testing (AST) may be important in predicting whether β-lactam treatment could be clinically beneficial in the setting of a host infection. For example, it is argued that determining the MIC in the presence of bicarbonate (HCO_3_) in the media may better reflect the host tissue milieu than standard MIC testing (50). β-lactams have sub-MIC favorable effects by weakening the bacterial cell wall and increasing toxin production, both of which lead to an enhanced host immune response and possibly faster clearance of the infection (55). β-lactams have been shown to “resensitize” MRSA to host defense peptides, leading to faster clearance of bacteremia (56). Anti-staphylococcal β-lactams can act synergistically with vancomycin, daptomycin (57), and host defense peptides (55). The MICs of β-lactams are also known to decrease when the MIC of glycol- and lipopeptides increase, a phenomenon termed the “seesaw effect” (58, 59). Finally, there is now clinical evidence that adjunctive β-lactams may enhance the clearance of *S. aureus* from the bloodstream in MRSA BSI. The CAMERA2 (Combination Antibiotics for Methicillin-Resistant *Staphylococcus aureus*) trial (60) reported that the rate of persistent bacteremia was lower by about half (11% vs 20%, *P* = 0.02) for participants with MRSA BSI who were randomly allocated to receive a combination of standard therapy (vancomycin or daptomycin) with adjunctive β-lactam compared to those who were allocated to standard therapy alone. It is unclear which, if any, of the above-mentioned mechanisms of β-lactams (Fig. 3) contributed to the shorter duration of bacteremia reported in the CAMERA2 trial. Unfortunately, the addition of an adjunctive β-lactam, which was mostly flucloxacillin or cloxacillin in CAMERA2, also increased toxicity, evidenced by a substantially higher risk of acute kidney injury (61). Using area under the curve (AUC) guided dosing of vancomycin (62), as well as combinations of other classes of β-lactam, such as cephalosporins or carbapenems, may limit the risk of nephrotoxicity based on pharmacological data (63), small case studies (64), and a small randomized trial (65).

Given the toxicity of adding β-lactams to the backbone anti-MRSA treatment, restricting the use of adjunctive β-lactams to MRSA strains that are more likely to respond to β-lactam therapy would seem wise. To this end, we need a better understanding of β-lactam susceptibility of MRSA strains. In this review, we discuss recently elucidated relationships between β-lactam susceptibility phenotypes and genotypes in MRSA strains, whether these phenotypes can predict response to treatment with adjunctive β-lactams, and highlight research gaps. Other, MIC-independent effects of β-lactams are not covered (for a recent review, see Sakoulas and Nizet [54]).

## β-LACTAM SUSCEPTIBILITY PHENOTYPES IN MRSA

CLSI (Clinical Laboratory Standards Institute) (66) and EUCAST (European Committee on Antimicrobial Susceptibility Testing) (67) suggest using a breakpoint for oxacillin (OX) of >2 mg/L or cefoxitin >4 mg/L (or ≤21 mm by disk diffusion), or *mecA* positivity by PCR to identify MRSA phenotypically and genotypically, respectively. Although all isolates satisfying these criteria are considered as MRSA, the β-lactam resistance phenotype varies considerably among MRSA isolates. The following sections will each address a β-lactam phenotype among *mecA*-positive *S. aureus*.

## OXACILLIN-SUSCEPTIBLE MRSA (OS-MRSA)

MRSA isolates harboring the *mecA* gene but testing susceptible to oxacillin (OX MIC ≤2 mg/L) on AST have been described (68–73). These isolates have been termed oxacillin-susceptible MRSA (OS-MRSA) or *mecA*-positive MSSA (74) and represent between 1.6% and 8.3% of *S. aureus* isolates in reports from human clinical isolates (70, 73, 75). The OS-MRSA phenotype raises concerns about potentially misdiagnosing MRSA (as defined by the presence of *mecA*) infections as MSSA using currently available phenotypic methods, and treating such infections with β-lactams, which theoretically might lead to treatment failure through induction of the *mecA* gene or selection of homoresistance (76). On the other hand, if these isolates are indeed more susceptible, β-lactam treatment may be effective as an adjunctive agent.

Clinical OS-MRSA strains may exhibit a β-lactam heteroresistant phenotype (43, 69, 77), where the majority of cells have low MIC to β-lactams, but a small minority express a high level of resistance at frequencies from 10^−9^ to 10^−5^ (77).

**Fig 4:**
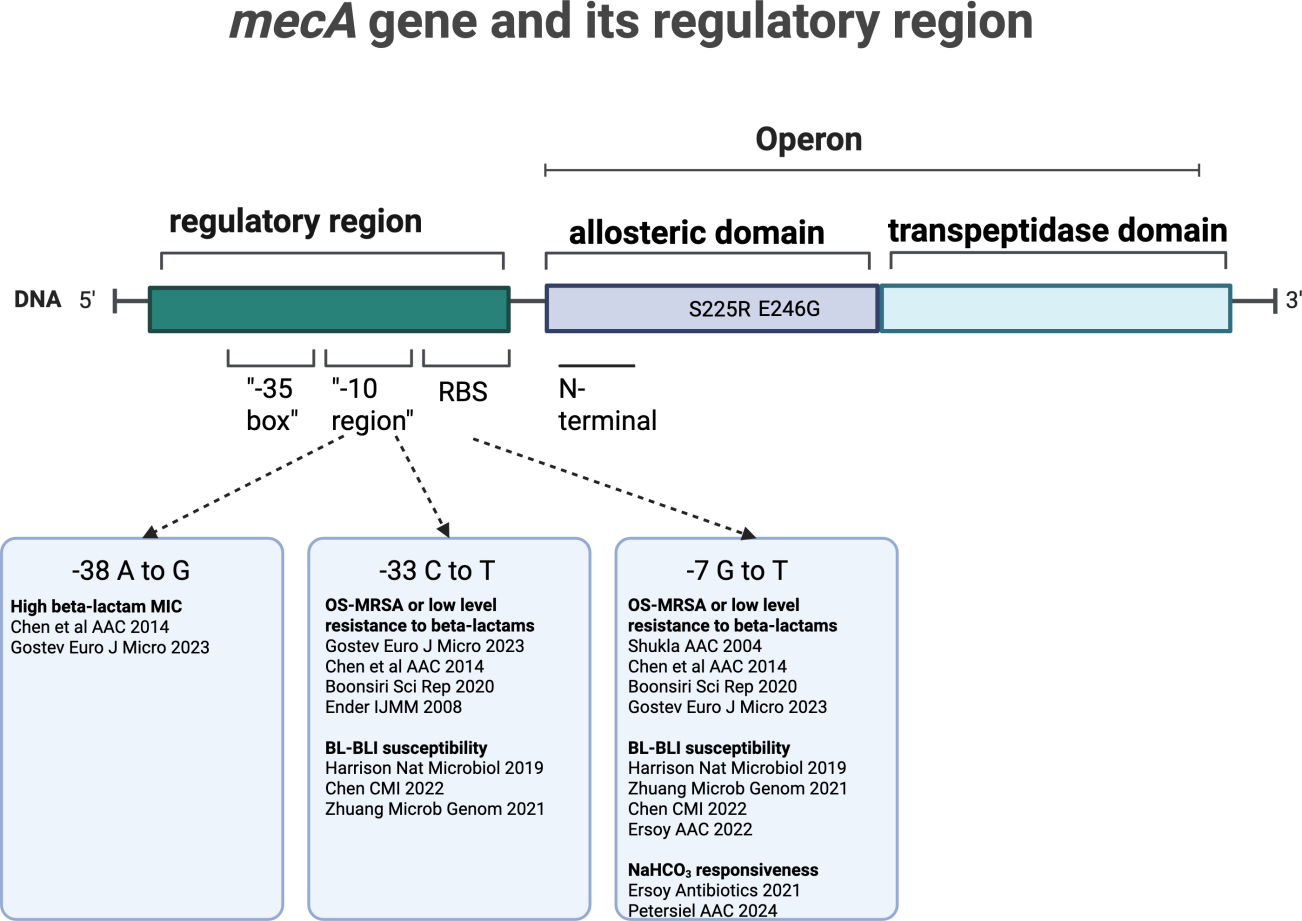
*mecA* gene and its regulatory region. Single-nucleotide polymorphisms (SNPs) reported to correlate with β-lactam resistance phenotypes are noted in the boxes (38, 42, 43, 49, 80–86). BL-BLI, beta-lactam–beta-lactamase inhibitor; MIC, minimum inhibitory concentration; OS-MRSA, oxacillin-susceptible methicillin-resistant *Staphylococcus aureus*; RBS, ribosome binding site.

Although originating from diverse genetic backgrounds, the heteroresistant OS-MRSA share some phenotypic and genotypic characteristics. They are often reported to produce PBP2a (42, 78), which leads to their correct diagnosis as MRSA by latex agglutination testing (79), but in some cases have been reported to have negative agglutination test results (77). In most reports, the OS-MRSA strains belong to clonal complexes (CCs) that are considered community-associated (CA) and harbor SCC*mec* types IV and V (42, 43, 69, 77), which contain a complete *mecA* gene (e.g., without premature stop codons), a truncated *mecR,* and absent *mecI* genes (32). Specific mutations in the *mecA* gene and its promoter have been frequently reported in OS-MRSA isolates (Fig. 4). Amino acid substitutions at positions 225 (S to R) and 246 (E to G) are the most commonly described (42, 43, 80, 81), but it is unclear whether they impact the OS-MRSA phenotype. Chen et al. (81) performed site-directed mutagenesis separately for these two amino acid substitutions and reported that it did not affect the oxacillin (OX) MIC. In the two largest collections of OS-MRSA from Japan (42) (*n* = 42) and Russia (43) (*n* = 60), all isolates carried single-nucleotide polymorphisms (SNPs) in the regulatory region of the *mecA* gene (Fig. 4) at position −7 (G to T substitution) in the ribosome binding site (RBS) or at position −33 (C to T substitution) in the “−10 box” which is the binding site of the *mecA* regulators, *mecI/blaI* and *mecR/blaR*. These two SNPs have been reported in other studies of MRSA exhibiting low-level resistance to β-lactams (38, 49, 80–83). The −7G to T and −33C to T substitutions have been shown to correlate with lower expression levels of *mecA* compared to isolates carrying −7G (38, 43, 81). To assess the role of *mecA* and its expression, Chen et al. (81) transformed different alleles of *mecA* and its promoter from clinical MRSA strains, including −7G to T and −33C to T, into MSSA strains from two genetic backgrounds (ST59 and ST6) and demonstrated that this was sufficient to achieve high-level oxacillin resistance in these backgrounds. In another experiment, Boonsiri et al. investigated a wider range of clinical OS-MRSA isolates and performed oxacillin resistance selection experiments. They found a wide range of mutations but not single-nucleotide polymorphisms (SNPs) in *mecA* or its promoter, suggesting that *mecA* mutations are not necessary for high-level resistance (42).

In rare cases, the OS-MRSA phenotype arises from an inactive PBP2a protein, typically due to a premature stop codon in the *mecA* gene (87, 88), transposon insertions (74), or through complete excision of the SCC*mec* cassette (89). In these cases, *in vitro* exposure to oxacillin selected for strains with an oxacillin-resistant phenotype with restored PBP2a function, either by correcting the amino acid sequence (74, 88), transposon excision or through stable re-integration of the SCC*mec* element (89).

*In vivo* data to support the use of β-lactams in infections caused by OS-MRSA are limited. Two studies have evaluated β-lactam monotherapy for OS-MRSA infection in a murine thigh infection model (90, 91). One of these studies compared β-lactam treatment (dicloxacillin) to vancomycin; dicloxacillin was less effective in 7/15 strains and as effective in 8/15 strains (91).

Data from clinical cases of OS-MRSA are mainly limited to case reports; hence, the best approach to treating such infections is unclear. Current recommendations are that any isolate testing positive for the *mecA* gene should be reported as MRSA, regardless of β-lactam MICs (66). Because published case reports (74, 76) focus on cases where the OS-MRSA phenotype became apparent due to PCR testing of the *mecA* gene following treatment failure, it is possible that some cases of OS-MRSA infections were misdiagnosed phenotypically as MSSA in the past and successfully treated with β-lactams.

Only one study addressed the vancomycin treatment outcomes of patients with OS-MRSA BSI. Jones et al. (92) compared the outcomes of 17 patients with OS-MRSA BSI to those of 17 matched patients with OR-MRSA BSI. As the diagnosis was confirmed by PCR, most patients in both groups were treated with vancomycin. Compared to OR-MRSA BSI, having an OS-MRSA BSI was associated with a need to change treatment due to lack of clinical response (59% vs 6%, *P* = 0.002) and a numerically higher rate of persistent BSI defined as ≥7 days of positive blood cultures (35% vs 12%, *P* = 0.2), but there was no difference in the median duration of bacteremia (3.5 days vs 3 days, *P* = 0.9). All-cause 30-day mortality was low at 6% and did not differ between the two groups (i.e., one patient died in each group). Whether adding a β-lactam to vancomycin (or daptomycin) actually leads to improved patient outcomes in infections caused by OS-MRSA is unknown and should be the focus of future research.

## SUSCEPTIBILITY TO β-LACTAM-β-LACTAMASE INHIBITORS COMBINATION (BL-BLI)

β-Lactamase inhibitors (BLI) were developed to counteract β-lactamase production by organisms such as *S. aureus*, which became prevalent soon after β-lactams were introduced (93). BL-BLI combinations are not considered clinically active in strains possessing PBP2a, which generally have low affinity for older generations of β-lactams. The affinity for PBP2a differs among β-lactams, with amoxicillin/ampicillin, penicillin, and imipenem having the highest affinity, and antistaphylococcal penicillins having the lowest (10). The relatively higher affinity of amoxicillin to PBP2a has led investigators to study combinations of penicillin and amoxicillin and a BLI for the treatment of MRSA infections.

Ba et al. (94) first reported that *mecC*-positive MRSA are susceptible *in vitro* to combinations of BL-BLI. The authors then tested *mecA*-positive MRSA strains (38) and reported that 213/298 isolates were phenotypically susceptible to the combination of penicillin and the β-lactamase inhibitor clavulanic acid, using an epidemiological cutoff (ECOFF) breakpoint ≤2 µg/mL. In another report, Ba et al. (95) showed that susceptibility to oxacillin and other β-lactams with activity against *S. aureus*, measured by disc diffusion assay, correlated well with the penicillin-clavulanate MIC, suggesting that these isolates are relatively more susceptible *in vitro* to a range of β-lactams with activity against MSSA. They reported that the BL-BLI susceptible phenotype of MRSA can be diagnosed by using the oxacillin disc diffusion assay with a cutoff of 10 mm, allowing for an easier and less costly method of diagnosis. Interestingly, the susceptible phenotype could only be revealed by using IsoSensitest Agar (ISA) but was “missed” by using the standard Mueller-Hinton Agar (MHA), suggesting that the media used for AST plays a role in exposing this phenotype. It is known that MHA is more sensitive than ISA for the detection of methicillin-resistance by disc diffusion (96), but the reasons for this difference are not clear.

Harrison et al. reported that the BL-BLI phenotypically “susceptible” isolates harbored SNPs in the promoter and coding region of the *mecA* gene, that could be used to annotate four “susceptible” genotypes (38). The most common SNPs were substitutions at positions −7 G to T and −33 C to T in the promoter and an E246G amino acid substitution in the allosteric domain of PBP2a (Fig. 4). These are the same promoter mutations reported from studies of OS-MRSA (42, 43, 80, 81), suggesting that the OS-MRSA and BL-BLI phenotypes likely share a core set of genotypes that are uncovered by different phenotypic assays. The promoter site mutations (−7G to T and −33 C to T) were significantly associated with reduced *mecA* expression as measured by reverse-transcription quantitative polymerase chain reaction (RT-qPCR) compared to isolates with −7G allele of *mecA*, while the mutation in the allosteric site led to increased affinity of PBP2a to penicillin-clavulanate. Furthermore, Ersoy et al. showed that a phenotypically BL-BLI-resistant MRSA isolate (CC5, penicillin-clavulanate MIC 12 mg/L and OX MIC 1,024 mg/L) became “susceptible” (penicillin-clavulanate MIC 0.25 mg/L and OX MIC 64 mg/L) when a point mutation was introduced in the *mecA* promoter at position −7 (a change from G to T) (84). On the other hand, Zhuang et al. reported that among a collection of MRSA isolates belonging to ST59, some genotypically “susceptible” (with a −7 G to T SNP) isolates were found to be phenotypically “resistant” and that the phenotype was a better predictor of the *mecA* expression levels than the genotype (82), implying that factors other than mutations in the *mecA* allele and promoter region also impact the β-lactam resistance level.

The BL-BLI “susceptible” genotype seems to be widespread geographically and linked to certain genetic markers such as sequence types (STs). For example, the rate of BL-BLI “susceptible” genotype was high among CA-MRSA strains such as those belonging ST59 from China (152/154, 98.7%)(49) or to the epidemic USA300 lineage (a common CA-MRSA belonging to ST8 from the United States [USA]) (457/457, 100%) (38), but it was low among STs that are considered healthcare associated (HA) such as ST22 from the United Kingdom (65/1,672, 3.8%) (38) and ST239 from China (0/116, 0%) (49).

The clinical applicability of these findings is still to be determined. Although there are no human studies that evaluated clinical response to BL-BLI treatment, some *in vivo* work has been done (38). Studies published from the 1980s to the early 1990s reported conflicting evidence regarding the effectiveness of BL-BLI treatment in MRSA infective endocarditis (IE) animal models (97–100). Cantoni et al. (98) showed that in an MRSA IE rat model (OX MIC 64 mg/L and amoxicillin-clavulanate MIC 16 mg/L), treatment with high-dose amoxicillin-clavulanate sterilized >90% of vegetations and did so more effectively than vancomycin (*P* = 0.02). Hirano and Bayer (100) similarly reported that in an MRSA IE rabbit model (OX MIC 64 mg/L), ampicillin-sulbactam was better at reducing vegetation bacterial densities than vancomycin (*P* < 0.005). Franciolli et al. (99) compared the efficacy of different β-lactams (cloxacillin, amoxicillin, and amoxicillin-clavulanate) to vancomycin for the treatment of IE in a rat model caused by β-lactamase-positive strains (cloxacillin MIC 32–64 mg/L, amoxicillin-clavulanate 8–16 mg/L, and amoxicillin 128 mg/L) and their β-lactamase-negative (plasmid cured) MRSA derivatives (cloxacillin 32–128 mg/L, amoxicillin-clavulanate 8 mg/L, and amoxicillin 8 mg/L). They found that for the β-lactamase-positive strains, amoxicillin-clavulanate was as good or even better at sterilizing vegetations than vancomycin and that cloxacillin was ineffective. For β-lactamase-negative strains (a minority in clinical infections), amoxicillin alone was more effective than all other β-lactams and vancomycin. On the other hand, Chambers et al. (101) reported that in an MRSA IE rabbit model (nafcillin MIC >64 mg/L, ampicillin-sulbactam 16 mg/L, and imipenem >64 mg/L), vancomycin was more effective in achieving sterile vegetation than any of the β-lactams tested (nafcillin, ticarcillin/clavulanate, ampicillin/sulbactam, and imipenem) (*P* = 0.006). In a second study by the same authors (97), ampicillin-sulbactam was inferior to vancomycin for the treatment of a homo-resistant MRSA strain (nafcillin MIC >128 mg/L and ampicillin-sulbactam 64 mg/L) or a hetero-resistant MRSA strain (nafcillin MIC 32 mg/L and ampicillin-sulbactam 32 mg/L) in a rabbit IE model. Significantly, the *in vivo* activity of the β-lactams in their study (101) correlated with both the binding affinity to PBP2a and the fraction of resistant cells within the bacterial population (i.e., the level of hetero-resistance to the specific β-lactam), suggesting that the choice of β-lactam and the bacterial strain are essential for predicting treatment outcomes. Heterogeneity in susceptibility patterns, treatment options, duration, doses, and choice of animal models makes a direct comparison between these studies limited. In general, studies showing benefit from BL-BLI treatment (98–100) used significantly higher doses of β-lactams (the amoxicillin component was given at 600–800 mg/kg daily) than studies reporting inferior outcomes with BL-BLI (amoxicillin component 150–300 mg/kg daily).

More recently, Harrison et al. reported the results of BL-BLI treatment of MRSA infections in a moth larvae and murine thigh models. In moth larvae infected by genetically “susceptible” MRSA strains (i.e., harboring a −7T to G SNP in the *mecA* promoter), treatment with ampicillin/penicillin-clavulanate resulted in increased survival over ampicillin or penicillin alone, but not over vancomycin. In larvae infected by genetically “resistant” strains, on the other hand, only vancomycin resulted in improved survival, and neither ampicillin/penicillin nor their combination with clavulanate resulted in any survival benefit over control. In the murine thigh model, an infection caused by a USA300 MRSA isolate with a BL-BLI susceptible genotype was treated with ampicillin in different doses, with or without clavulanic acid, vancomycin, or saline. Treatment with high-dose ampicillin-clavulanate (100/20 mg/kg) reduced bacterial loads significantly and similarly (*P* = NS) to vancomycin (40 mg/kg). In contrast, treatment with ampicillin alone or treatment with lower doses of ampicillin-clavulanate (10/2 or 30/6 mg/kg) showed no benefit and resulted in similar bacterial loads as the control.

Taken together, these data support a possible role for adjunctive BL-BLI treatment for certain MRSA isolates, such as those with a favorable genetic background and which harbor certain *mecA* alleles, but further studies are needed before any treatment recommendations are made. It will also be interesting to investigate whether BL-BLI sensitivity is predictive of treatment response only to BL-BLI or also to other anti-staphylococcal β-lactams such as the antistaphylococcal penicillins and first-generation cephalosporins.

## BICARBONATE (NaHCO_3_) RESPONSIVENESS

NaHCO_3_ responsiveness is a β-lactam susceptibility phenotype, where some MRSA strains exhibit lower MICs (fourfold decrease) to cefazolin and/or oxacillin when measured on Mueller Hinton broth (MHB) in the presence of NaHCO_3_ compared to standard MHB (52). The NaHCO_3_ supplemented media is meant to better reflect conditions at the site of infection (50). Ersoy et al. tested five MRSA strains and showed that the MIC of cefazolin and oxacillin was more than fourfold lower (and below the clinical breakpoint) for 2 of these strains when performed on MHB supplemented by 44 nM NaHCO_3_ compared to standard MHB (52). Importantly, the lower MICs measured on NaHCO_3_ supplemented media predicted tissue bacterial clearance with cefazolin and oxacillin treatments (compared to control) in a rabbit endocarditis model (52) and in a simulated *ex vivo* endocardial vegetations model (102).

The prevalence of the NaCHO_3_ responsive phenotype differs geographically and is likely related to the genetic background of the strain. In a collection of 58 clinical MRSA strains from the USA, 76% (44/58) and 36% (21/58) were cefazolin- and oxacillin-NaHCO_3_ responsive, respectively (103). The rate of oxacillin-NaHCO_3_ responsiveness was higher among isolates belonging to CC8 (a common CA-MRSA clone from the USA) compared to other CCs; 59% (13/22) of isolates in CC8 were oxacillin-NaHCO_3_ responsive. The authors did not find a similar correlation with the cefazolin-NaHCO_3_ responsive phenotype among other CCs. Petersiel et al. tested 117 MRSA BSI isolates from Australia, New Zealand, Singapore, and Israel (85) and found 31% (36/117) and 25% (21/117) to be cefazolin- and oxacillin-NaHCO_3_ responsive, respectively. Further supporting the impact of clonal effects on this phenotype, the rate of NaHCO_3_ responsiveness was higher among isolates belonging to ST93 (an Australian CA-MRSA strain), where 57% (20/35) and 38% (9/24) of isolates were cefazolin- and oxacillin-NaHCO_3_ responsive, respectively. By contrast, the rate of NaHCO3 responsiveness in ST 22 (a common HA-MRSA strain) was 18% (7/38) and 7% (2/28) for cefazolin and oxacillin, respectively.

The *mecA* allele has also been shown to correlate with the NaHCO_3_-responsive phenotype (Fig. 4). Ersoy et al. reported that a *mecA* allele with a promoter substitution at position −7 from G to T had a good sensitivity of 93.3% but a specificity of only 66.7% in predicting the NaHCO_3_-responsive phenotype (86). Petersiel et al. also reported an association between the NaHCO_3_-responsive phenotype and SNPs in the promoter of *mecA* (85). Compared to isolates without SNPs in the promoter or coding region of *mecA,* isolates who had a single SNP at position −7G to T (annotated as “susceptible 2” allele) or a combination of three SNPs, −38 A to G, −33 C to T, and −7 G to T (annotated as “susceptible 5” allele) were more likely to be cefazolin-NaHCO_3_ responsive (“susceptible 2” OR 4.6, 95% CI 1.5–14), “susceptible 5” OR 20.8, 95% CI 3.4–128.5), or oxacillin-NaHCO_3_ responsive (“susceptible 2” OR 13.2, 95% CI 1.6–111), “susceptible 5” OR 60, 95% CI 4.5–797). As noted above, the −7 G to T and −33 C to T SNPs had been reported from studies of OS-MRSA (42, 43) and BL-BLI susceptible isolates (38, 49, 82). To investigate the effect of the −7G/T and 246E/G on the NaHCO_3_ phenotype, Ersoy et al. (84) constructed isogenic mutants in two genetic backgrounds, a NaHCO_3_-responsive and a non-responsive strain. The NaHCO_3_ responsive strain became non-responsive when a −7G point mutation (resistant genotype) was introduced, while the NaHCO_3_ non-responsive strain remained non-responsive even with a −7G to T mutation (susceptible genotype). This suggests that there are factors in addition to the *mecA* and promoter alleles that determine the NaHCO_3_ responsiveness of a given strain. Ersoy et al. (104) conducted a genome-wide association study (GWAS) on 103 MRSA strains from skin and soft tissue infections from the USA to search for mutations correlating with the NaHCO_3_-responsive phenotype. The authors reported that missense mutations in a gene they termed *sabR*, an *araC* family transcriptional regulator, were associated with the NaHCO_3_ non-responsive phenotype. Deletion of the gene from an NaHCO_3_-responsive strain rendered the strain non-responsive. However, complementation of the gene was unable to restore the responsive phenotype.

The mechanism behind NaHCO_3_ re-sensitization to β-lactams is not entirely understood, and several biochemical and genetic pathways have been explored. In the presence of NaHCO_3_, the “responsive” strains showed repression of several genes involved in virulence, membrane, and cell wall synthesis, compared to the “non-responsive” strains (105). These repressed genes included *blaZ*, *mecA* (106), the virulence regulator *agr* (105), *pbp4* (106), which is needed for peptidoglycan cross-linking in the presence of oxacillin, and *sarA* and *vraSR* (both play a role in the “see-saw” effect of daptomycin). Similarly, certain proteins are downregulated in the presence of NaHCO,_3_ such as PrsA (a chaperone for PBP2a) (106), PBP2a (52), and wall teichoic acid (a key factor in the cell wall that acts as a scaffold for PBP2a) (107).

The clinical relevance of the NaHCO3-responsive phenotype is unclear. Petersiel et al. investigated an association between the NaHCO_3_ responsive phenotype and outcomes of patients with MRSA bacteremia (*n* = 68) (85) who were allocated to receive a combination of standard therapy with a β-lactam (anti-staphylococcal penicillin or a 1st generation cephalosporin) as part of the CAMERA2 randomized trial. The rate of persistent bacteremia on trial day 5 was similar for patients treated with a β-lactam regardless of whether the isolate was NaHCO_3_ responsive or not (9% vs 11% in cefazolin-NaHCO_3_ responsive compared to non-responsive strains (*P* = 0.82), and 12% vs 10% in oxacillin-NaHCO_3_ responsive compared to non-responsive strains (*P* = 0.81)), leading the authors to conclude that treatment with β-lactams of infections caused by NaHCO_3_ responsive strains in this specific clinical trial was not associated with a better clinical outcome. The observation that the beneficial effect of β-lactams in reducing the duration of bacteremia does not correlate with susceptibility to β-lactams may suggest that β-lactams work to enhance immune-mediated clearance. Studying the effect of bacterial factors on clinical outcomes is challenging in the setting of small data sets such as the one mentioned above because outcomes such as persistence and mortality are mainly attributed to host risk factors (e.g., comorbidities and source of infection) (108), and these dominant factors might mask the impact of bacterial factors (109). Large-scale studies, including detailed metadata, phenotypic and genotypic data, are necessary to delineate the contribution of bacterial factors such as phenotypes or genetic determinants on clinical outcomes.

## SUSCEPTIBILITY TO β-LACTAMS COMBINATIONS

Another interesting phenotype of MRSA susceptibility to β-lactams is that some MRSA strains are inhibited *in vitro* by combinations of β-lactams that target different penicillin-binding proteins (PBP) (12, 15, 110–114). The strategy of combining two β-lactams has been used with enterococcal infections, which are relatively resistant or tolerant to β-lactams. The combination of ceftriaxone with ampicillin is used to overcome this tolerance by targeting several PBPs simultaneously, where ceftriaxone binds to PBP2 and PBP3, and ampicillin binds to PBP4 and PBP5 (115). In contrast to enterococcal infections, this strategy has not been extensively explored for MRSA.

Several β-lactams combinations that target two or more PBPs have been described to act synergistically in MRSA:

### Cephalosporins in combination with other β-lactams

Cefoxitin was the most frequently studied combination. It selectively inhibits PBP4, which has been shown to promote β-lactam resistance in CA-MRSA but not in HA-MRSA strains. Memmi et al. (110) reported that in the CA-MRSA MW2 (ST1 SCC*mec* IV) and USA300 (ST8, SCC*mec* IV), but not in the HA-MRSA COL (ST250, SCC*mec* I), N315 (ST5 SCC*mec* II), and Mu50 (ST5 SCC*mec* II), ∆PBP4 knock-out mutants had 16-fold lower MIC to oxacillin than the wild type (WT). The authors found that the CA-MRSA ∆PBP4 mutants had a lower level of highly cross-linked peptidoglycan and diminished transcription of PBP2. Surprisingly, even though the oxacillin MIC of the ∆PBP4 was lower, the *mecA* transcription of this mutant was unchanged from the WT. They showed that for 30 CA-MRSA clinical skin and soft tissue infections strains harboring SCC*mec* IV, the MIC of oxacillin decreased by 64-fold to below the clinical breakpoint (from a median of 64 to <1 mg/L) in the presence of 0.25 × MIC of cefoxitin, compared to an 8-fold decrease that was observed for 30 HA-MRSA strains (from a median of 256 to 32 mg/L). Quach et al. (113) reported that MRSA strains cluster into two distinctive groups based on their degree of lysis when exposed to oxacillin, and that strains that had a high degree of lysis were closely related to the CA-MRSA strain USA300. The high-lysis strains responded synergistically, as determined by a checkerboard assay, to a combination of oxacillin and cefoxitin administered at physiologically attainable levels. However, genotypic markers alone could not predict the high lysis phenotype or presence of synergy, as some strains that were closely related to USA300 had a low lysis phenotype and showed inconsistent results in the checkerboard assays. Banerjee et al. (112) used time-kill assays to test for synergy. They reported that the combination of cefoxitin with either cefazolin, cefuroxime, or nafcillin was synergistic *in vitro* (>2 log_10_ reduction in CFU/mL for the combination compared to the most active agent alone) in CA-MRSA strains harboring a SCC*mec*IV (ST8IV and ST1IV), but not in HA-MRSA strains (COL and N315 harboring SCC*mec*I and II, respectively). The synergy was most pronounced early in the growth and with low inoculum (10^4.5^–10^6.5^ CFU/mL). The reason for the discrepancy between HA-MRSA and CA-MRSA response to cefoxitin combinations is unclear. It was speculated that this was related to the SCC*mec* cassette type until Reichmann and Pinho (114) exchanged the SCC*mec* in COL (a HA-MRSA) from SCC*mec*I to SCC*mec*IV (from MW2, a CA-MRSA) and found that this swap did not affect oxacillin MIC in the presence of cefoxitin. They concluded that genetic factors outside the SCC*mec* cassette determine the synergistic activity of cefoxitin and oxacillin. Finally, the combination of cefdinir, targeting PBP2 and PBP3, and amoxicillin, targeting PBP1, PBP2, and PBP3, was shown to be synergistic by checkerboard assay against diverse *S. aureus* isolates representing both hospital- and community-associated MRSA strains, as well as MSSA (15). The authors also reported that in time-kill assays, the combination was synergistic against both USA300 and COL (representing CA-MRSA and HA-MRSA, respectively), and that it offered survival benefit over each of the antibiotics alone in *G. mellonella* (larvae) infected with these two MRSA strains.

### Carbapenems in combination with other β-lactams

Meropenem, ertapenem, and imipenem bind most strongly to PBP1 (111), 2 (7), and 1–3 (7), respectively, in *S. aureus*. Harrison et al. (38) have reported that the susceptibility of MRSA strains to carbapenems varies and correlates with the strain’s susceptibility to other antistaphylococcal β-lactams and the *mecA* genotype. In the early 1990s, Sumita and Mitsuhashi (116) classified MRSA isolates from Japan as low-resistance-MRSA and high-resistance-MRSA according to their MIC to meropenem (<6.25 and >12.5 mg/L respectively) and showed that for low-resistance-MRSA, but not high-resistance-MRSA isolates, the combination of meropenem (or imipenem) with cefazolin was synergistic when assessed by the checkerboard method. Other carbapenem-cephalosporin combinations were synergistic against both low- and high-resistance MRSA. More recently, Gonzales et al. (111) demonstrated *in vitro* synergy by checkerboard assay of the combination of meropenem (or imipenem) and piperacillin and tazobactam at a 1:1:1 ratio against HA-MRSA N315 strain. They speculated that the mechanism involves allosteric change in PBP2a by meropenem that renders it accessible for the other β-lactam to bind, together with binding of meropenem to PBP1 and piperacillin to PBP2, and tazobactam protection from β-lactamase. They showed that this combination was not synergistic against MSSA, supporting a role of PBP2a in the synergy of these three drugs. Yoneda et al. (117) tested 121 MRSA strains from the USA, mostly from two genetic backgrounds: USA100 (CC5, SCC*mec* type II) and USA300 (CC8, SCC*mec* type IV), and reported that susceptibility to meropenem (but not piperacillin) correlated with susceptibility to this triple combination. The SCC*mec* type also correlated with susceptibility to this combination, where isolates harboring SCC*mec*IV were more susceptible than isolates with SCC*mec*II. The meropenem/piperacillin/tazobactam combination (VIO-001) was tested in a rabbit central line model using a USA300 MRSA isolate and showed comparable results to vancomycin in clearing the bloodstream infection and eradicating the infection from deep tissues (51). In a neutropenic mouse model infected by an N315 MRSA strain, blood cultures drawn 11 hours after infection onset were negative in mice treated with either linezolid, meropenem/piperacillin/tazobactam, or meropenem/piperacillin, but not in mice treated with meropenem, piperacillin, or piperacillin/tazobactam (111). This finding suggests that a lower carbapenem resistance level does not fully explain the clinical response to the meropenem/piperacillin/tazobactam combination, and that synergy between β-lactams likely contributes.

The combination of cefazolin with ertapenem was also shown to be synergistic against MSSA and two strains of CA-MRSA (MW2 and USA300), but not a HA-MRSA (USA200), when determined by a checkerboard assay (12). The authors then assessed 15 other MRSA isolates for synergy using a disc diffusion assay and found 67% of them showed synergy (no details regarding these strains were provided). Clinical evidence regarding the benefit of the combination of ertapenem and cefazolin comes from case reports and small case series where the combination was given as salvage therapy to patients with persistent MSSA bacteremia (118, 119). The ongoing *Staphylococcus aureus* Network Adaptive Platform (SNAP) trial is enrolling patients into a phase 2 study that compares cefazolin monotherapy to the combination of cefazolin and ertapenem for MSSA bacteremia (ClinicalTrials.gov ID NCT04886284). No reports of using the cefazolin-ertapenem combination to treat MRSA infections have been published to date.

## CONCLUSIONS

Collectively, the data presented here highlight that even though MRSA is considered inherently resistant to β-lactams, some strains exhibit increased *in vitro* susceptibility to β-lactams, while others do not. Increased susceptibility to β-lactams is more common among CA-MRSA strains, but there is considerable heterogeneity. The mechanisms behind the increased susceptibility are complex and related to both the genetic background of the strain and to mutations in genes involved in cell-wall metabolism. Several phenotypes may assist in identifying these so-called susceptible strains that could potentially be treated with a combination containing a β-lactam. A targeted approach, where combinations containing β-lactams are used to treat these “susceptible” infections, but not used for “resistant” infections, may prevent unnecessary toxicity.

Future studies should attempt to better define these β-lactam-susceptible strains, how these different β-lactam phenotypes overlap, and the genetic basis underlying low-level β-lactam resistance beyond the *mecA* genotype. Ideally, the β-lactam susceptibility phenotype could then be incorporated in a randomized clinical trial which would assess whether adding a β-lactam to treat β-lactam-“susceptible” MRSA infections improves patient outcomes.

## References

[1] Bai AD, Lo CKL, Komorowski AS, Suresh M, Guo K, Garg A, Tandon P, Senecal J, Del Corpo O, Stefanova I, Fogarty C, Butler-Laporte G, McDonald EG, Cheng MP, Morris AM, Loeb M, Lee TC. 2022. Staphylococcus aureus bacteraemia mortality: a systematic review and meta-analysis. Clin Microbiol Infect 28:1076–1084. doi:10.1016/j.cmi.2022.03.015 35339678

[2] Cosgrove SE, Sakoulas G, Perencevich EN, Schwaber MJ, Karchmer AW, Carmeli Y. 2003. Comparison of mortality associated with methicillin-resistant and methicillin-susceptible Staphylococcus aureus bacteremia: a meta-analysis. Clin Infect Dis 36:53–59. doi:10.1086/345476 12491202

[3] Stevens DL. 2006. The role of vancomycin in the treatment paradigm. Clin Infect Dis 42:S51–S57. doi:10.1086/491714 16323121

[4] van Hal SJ, Jensen SO, Vaska VL, Espedido BA, Paterson DL, Gosbell IB. 2012. Predictors of mortality in Staphylococcus aureus bacteremia. Clin Microbiol Rev 25:362–386. doi:10.1128/CMR.05022-11 22491776 PMC3346297

[5] Peacock SJ, Paterson GK. 2015. Mechanisms of methicillin resistance in Staphylococcus aureus. Annu Rev Biochem 84:577–601. doi:10.1146/annurev-biochem-060614-034516 26034890

[6] Berti AD, Sakoulas G, Nizet V, Tewhey R, Rose WE. 2013. β-Lactam antibiotics targeting PBP1 selectively enhance daptomycin activity against methicillin-resistant Staphylococcus aureus. Antimicrob Agents Chemother 57:5005–5012. doi:10.1128/AAC.00594-13 23896478 PMC3811465

[7] Ferrer-González E, Kaul M, Parhi AK, LaVoie EJ, Pilch DS. 2017. β-Lactam antibiotics with a high affinity for PBP2 act synergistically with the FtsZ-targeting agent TXA707 against methicillin-resistant Staphylococcus aureus. Antimicrob Agents Chemother 61:e00863-17. doi:10.1128/AAC.00863-17 28630190 PMC5571351

[8] Dumitrescu O, Choudhury P, Boisset S, Badiou C, Bes M, Benito Y, Wolz C, Vandenesch F, Etienne J, Cheung AL, Bowden MG, Lina G. 2011. β-Lactams interfering with PBP1 induce Panton-Valentine leukocidin expression by triggering sarA and rot global regulators of Staphylococcus aureus. Antimicrob Agents Chemother 55:3261–3271. doi:10.1128/AAC.01401-10 21502633 PMC3122422

[9] Georgopapadakou NH, Liu FY. 1980. Binding of beta-lactam antibiotics to penicillin-binding proteins of Staphylococcus aureus and Streptococcus faecalis: relation to antibacterial activity. Antimicrob Agents Chemother 18:834–836. doi:10.1128/AAC.18.5.834 6778388 PMC284100

[10] Chambers HF, Sachdeva M. 1990. Binding of β-lactam antibiotics to penicillin-binding proteins in methicillin-resistant Staphylococcus aureus. J Infect Dis 161:1170–1176. doi:10.1093/infdis/161.6.1170 2345297

[11] Farha MA, Leung A, Sewell EW, D’Elia MA, Allison SE, Ejim L, Pereira PM, Pinho MG, Wright GD, Brown ED. 2013. Inhibition of WTA synthesis blocks the cooperative action of PBPs and sensitizes MRSA to β-lactams. ACS Chem Biol 8:226–233. doi:10.1021/cb300413m 23062620 PMC3552485

[12] Sakoulas G, Olson J, Yim J, Singh NB, Kumaraswamy M, Quach DT, Rybak MJ, Pogliano J, Nizet V. 2016. Cefazolin and ertapenem, a synergistic combination used to clear persistent Staphylococcus aureus bacteremia. Antimicrob Agents Chemother 60:6609–6618. doi:10.1128/AAC.01192-16 27572414 PMC5075066

[13] Kosowska-Shick K, McGhee PL, Appelbaum PC. 2010. Affinity of ceftaroline and other β-lactams for penicillin-binding proteins from Staphylococcus aureus and Streptococcus pneumoniae. Antimicrob Agents Chemother 54:1670–1677. doi:10.1128/AAC.00019-10 20194704 PMC2863635

[14] Davies TA, Page MGP, Shang W, Andrew T, Kania M, Bush K. 2007. Binding of ceftobiprole and comparators to the penicillin-binding proteins of Escherichia coli, Pseudomonas aeruginosa, Staphylococcus aureus, and Streptococcus pneumoniae. Antimicrob Agents Chemother 51:2621–2624. doi:10.1128/AAC.00029-07 17470659 PMC1913263

[15] Altarawneh H, Alhomra T, Alharbi M, Fan Y, Derrick JP, Xia G. 2024. Synergistic bactericidal activity of a novel dual β-lactam combination against methicillin-resistant Staphylococcus aureus. J Antimicrob Chemother 79:1677–1682. doi:10.1093/jac/dkae165 38831599 PMC11215534

[16] Yang Y, Bhachech N, Bush K. 1995. Biochemical comparison of imipenem, meropenem and biapenem: permeability, binding to penicillin-binding proteins, and stability to hydrolysis by β-lactamases. J Antimicrob Chemother 35:75–84. doi:10.1093/jac/35.1.75 7768785

[17] Georgopapadakou NH, Smith SA, Bonner DP. 1982. Penicillin-binding proteins in a Staphylococcus aureus strain resistant to specific β-lactam antibiotics. Antimicrob Agents Chemother 22:172–175. doi:10.1128/AAC.22.1.172 7125630 PMC183698

[18] Truesdell SE, Zurenko GE, Laborde AL. 1989. Interaction of cephalosporins with penicillin-binding proteins of methicillin-resistant Staphylococcus aureus. J Antimicrob Chemother 23:13–19. doi:10.1093/jac/23.suppl_d.13 2722720

[19] Georgopapadakou NH, Smith SA, Cimarusti CM, Sykes RB. 1983. Binding of monobactams to penicillin-binding proteins of Escherichia coli and Staphylococcus aureus: relation to antibacterial activity. Antimicrob Agents Chemother 23:98–104. doi:10.1128/AAC.23.1.98 6338822 PMC184624

[20] Otero LH, Rojas-Altuve A, Llarrull LI, Carrasco-López C, Kumarasiri M, Lastochkin E, Fishovitz J, Dawley M, Hesek D, Lee M, Johnson JW, Fisher JF, Chang M, Mobashery S, Hermoso JA. 2013. How allosteric control of Staphylococcus aureus penicillin binding protein 2a enables methicillin resistance and physiological function. Proc Natl Acad Sci USA 110:16808–16813. doi:10.1073/pnas.1300118110 24085846 PMC3800995

[21] Holland TL, Cosgrove SE, Doernberg SB, Jenkins TC, Turner NA, Boucher HW, Pavlov O, Titov I, Kosulnykov S, Atanasov B, Poromanski I, Makhviladze M, Anderzhanova A, Stryjewski ME, Assadi Gehr M, Engelhardt M, Hamed K, Ionescu D, Jones M, Saulay M, Smart J, Seifert H, Fowler VG Jr, ERADICATE Study Group. 2023. Ceftobiprole for treatment of complicated Staphylococcus aureus bacteremia. N Engl J Med 389:1390–1401. doi:10.1056/NEJMoa2300220 37754204

[22] File TM Jr, Wilcox MH, Stein GE. 2012. Summary of ceftaroline fosamil clinical trial studies and clinical safety. Clin Infect Dis 55:S173–S180. doi:10.1093/cid/cis559 22903949

[23] Long SW, Olsen RJ, Mehta SC, Palzkill T, Cernoch PL, Perez KK, Musick WL, Rosato AE, Musser JM. 2014. PBP2a mutations causing high-level ceftaroline resistance in clinical methicillin-resistant Staphylococcus aureus isolates. Antimicrob Agents Chemother 58:6668–6674. doi:10.1128/AAC.03622-14 25155594 PMC4249384

[24] Becker K, van Alen S, Idelevich EA, Schleimer N, Seggewiß J, Mellmann A, Kaspar U, Peters G. 2018. Plasmid-encoded transferable mecB-mediated methicillin resistance in Staphylococcus aureus. Emerg Infect Dis 24:242–248. doi:10.3201/eid2402.171074 29350135 PMC5782906

[25] Paterson GK, Harrison EM, Holmes MA. 2014. The emergence of mecC methicillin-resistant Staphylococcus aureus. Trends Microbiol 22:42–47. doi:10.1016/j.tim.2013.11.003 24331435 PMC3989053

[26] Hackbarth CJ, Chambers HF. 1993. blaI and blaR1 regulate beta-lactamase and PBP 2a production in methicillin-resistant Staphylococcus aureus. Antimicrob Agents Chemother 37:1144–1149. doi:10.1128/AAC.37.5.1144 8517704 PMC187918

[27] Niemeyer DM, Pucci MJ, Thanassi JA, Sharma VK, Archer GL. 1996. Role of mecA transcriptional regulation in the phenotypic expression of methicillin resistance in Staphylococcus aureus. J Bacteriol 178:5464–5471. doi:10.1128/jb.178.18.5464-5471.1996 8808937 PMC178368

[28] Katayama Y, Ito T, Hiramatsu K. 2000. A new class of genetic element, staphylococcus cassette chromosome mec, encodes methicillin resistance in Staphylococcus aureus. Antimicrob Agents Chemother 44:1549–1555. doi:10.1128/AAC.44.6.1549-1555.2000 10817707 PMC89911

[29] Hartman BJ, Tomasz A. 1984. Low-affinity penicillin-binding protein associated with beta-lactam resistance in Staphylococcus aureus. J Bacteriol 158:513–516. doi:10.1128/jb.158.2.513-516.1984 6563036 PMC215458

[30] International Working Group on the Classification of Staphylococcal Cassette Chromosome Elements (IWG-SCC). 2009. Classification of staphylococcal cassette chromosome mec (SCCmec): guidelines for reporting novel SCCmec elements. Antimicrob Agents Chemother 53:4961–4967. doi:10.1128/AAC.00579-09 19721075 PMC2786320

[31] Wang W, Hu Y, Baker M, Dottorini T, Li H, Dong Y, Bai Y, Fanning S, Li F. 2022. Novel SCCmec type XV (7A) and two pseudo-SCCmec variants in foodborne MRSA in China. J Antimicrob Chemother 77:903–909. doi:10.1093/jac/dkab500 35040979

[32] Lakhundi S, Zhang K. 2018. Methicillin-resistant Staphylococcus aureus: molecular characterization, evolution, and epidemiology. Clin Microbiol Rev 31:e00020-18. doi:10.1128/CMR.00020-18 30209034 PMC6148192

[33] Panchal VV, Griffiths C, Mosaei H, Bilyk B, Sutton JAF, Carnell OT, Hornby DP, Green J, Hobbs JK, Kelley WL, Zenkin N, Foster SJ. 2020. Evolving MRSA: high-level β-lactam resistance in Staphylococcus aureus is associated with RNA polymerase alterations and fine tuning of gene expression. PLoS Pathog 16:e1008672. doi:10.1371/journal.ppat.1008672 32706832 PMC7380596

[34] Mwangi MM, Kim C, Chung M, Tsai J, Vijayadamodar G, Benitez M, Jarvie TP, Du L, Tomasz A. 2013. Whole-genome sequencing reveals a link between β-lactam resistance and synthetases of the alarmone (p)ppGpp in Staphylococcus aureus. Microb Drug Resist 19:153–159. doi:10.1089/mdr.2013.0053 23659600 PMC3662374

[35] Tomasz A, Nachman S, Leaf H. 1991. Stable classes of phenotypic expression in methicillin-resistant clinical isolates of staphylococci. Antimicrob Agents Chemother 35:124–129. doi:10.1128/AAC.35.1.124 2014967 PMC244952

[36] Sutherland R, Rolinson GN. 1964. Characteristics of methicillin-resistant staphylococci. J Bacteriol 87:887–899. doi:10.1128/jb.87.4.887-899.1964 14137628 PMC277108

[37] Ender M, McCallum N, Adhikari R, Berger-Bächi B. 2004. Fitness cost of SCCmec and methicillin resistance levels in Staphylococcus aureus. Antimicrob Agents Chemother 48:2295–2297. doi:10.1128/AAC.48.6.2295-2297.2004 15155238 PMC415608

[38] Harrison EM, Ba X, Coll F, Blane B, Restif O, Carvell H, Köser CU, Jamrozy D, Reuter S, Lovering A, Gleadall N, Bellis KL, Uhlemann AC, Lowy FD, Massey RC, Grilo IR, Sobral R, Larsen J, Rhod Larsen A, Vingsbo Lundberg C, Parkhill J, Paterson GK, Holden MTG, Peacock SJ, Holmes MA. 2019. Genomic identification of cryptic susceptibility to penicillins and β-lactamase inhibitors in methicillin-resistant Staphylococcus aureus. Nat Microbiol 4:1680–1691. doi:10.1038/s41564-019-0471-0 31235959 PMC7611363

[39] Bilyk BL, Panchal VV, Tinajero-Trejo M, Hobbs JK, Foster SJ. 2022. An Interplay of Multiple Positive and Negative Factors Governs Methicillin Resistance in Staphylococcus aureus. Microbiol Mol Biol Rev 86:e0015921. doi:10.1128/mmbr.00159-21 35420454 PMC9199415

[40] Kim C, Mwangi M, Chung M, Milheirço C, de Lencastre H, Tomasz A. 2013. The mechanism of heterogeneous beta-lactam resistance in MRSA: key role of the stringent stress response. PLoS One 8:e82814. doi:10.1371/journal.pone.0082814 24349368 PMC3857269

[41] Dordel J, Kim C, Chung M, Pardos de la Gándara M, Holden MTJ, Parkhill J, de Lencastre H, Bentley SD, Tomasz A. 2014. Novel determinants of antibiotic resistance: identification of mutated loci in highly methicillin-resistant subpopulations of methicillin-resistant Staphylococcus aureus. mBio 5:e01000. doi:10.1128/mBio.01000-13 24713324 PMC3993859

[42] Boonsiri T, Watanabe S, Tan X-E, Thitiananpakorn K, Narimatsu R, Sasaki K, Takenouchi R, Sato’o Y, Aiba Y, Kiga K, Sasahara T, Taki Y, Li F-Y, Zhang Y, Azam AH, Kawaguchi T, Cui L. 2020. Identification and characterization of mutations responsible for the β-lactam resistance in oxacillin-susceptible mecA-positive Staphylococcus aureus. Sci Rep 10:16907. doi:10.1038/s41598-020-73796-5 33037239 PMC7547103

[43] Gostev V, Sabinova K, Sopova J, Kalinogorskaya O, Sulian O, Chulkova P, Velizhanina M, Pavlova P, Danilov L, Kraeva L, Polev D, Martens E, Sidorenko S. 2023. Phenotypic and genomic characteristics of oxacillin-susceptible mecA-positive Staphylococcus aureus, rapid selection of high-level resistance to beta-lactams. Eur J Clin Microbiol Infect Dis 42:1125–1133. doi:10.1007/s10096-023-04646-1 37515660

[44] Aiba Y, Katayama Y, Hishinuma T, Murakami-Kuroda H, Cui L, Hiramatsu K. 2013. Mutation of RNA polymerase β-subunit gene promotes heterogeneous-to-homogeneous conversion of β-lactam resistance in methicillin-resistant Staphylococcus aureus. Antimicrob Agents Chemother 57:4861–4871. doi:10.1128/AAC.00720-13 23877693 PMC3811421

[45] Sommer A, Fuchs S, Layer F, Schaudinn C, Weber RE, Richard H, Erdmann MB, Laue M, Schuster CF, Werner G, Strommenger B. 2021. Mutations in the gdpP gene are a clinically relevant mechanism for β-lactam resistance in meticillin-resistant Staphylococcus aureus lacking mec determinants. Microb Genom 7. doi:10.1099/mgen.0.000623 PMC8715439 34486969

[46] Giulieri SG, Guérillot R, Kwong JC, Monk IR, Hayes AS, Daniel D, Baines S, Sherry NL, Holmes NE, Ward P, Gao W, Seemann T, Stinear TP, Howden BP. 2020. Comprehensive genomic investigation of adaptive mutations driving the low-level oxacillin resistance phenotype in Staphylococcus aureus. mBio 11:e02882-20. doi:10.1128/mBio.02882-20 33293382 PMC7733948

[47] McDougal LK, Thornsberry C. 1986. The role of beta-lactamase in staphylococcal resistance to penicillinase-resistant penicillins and cephalosporins. J Clin Microbiol 23:832–839. doi:10.1128/jcm.23.5.832-839.1986 3011847 PMC268732

[48] Liu C, Bayer A, Cosgrove SE, Daum RS, Fridkin SK, Gorwitz RJ, Kaplan SL, Karchmer AW, Levine DP, Murray BE, Rybak MJ, Talan DA, Chambers HF. 2011. Clinical practice guidelines by the infectious diseases society of america for the treatment of methicillin-resistant Staphylococcus aureus infections in adults and children. Clin Infect Dis 52:e18–e55. doi:10.1093/cid/ciq146 21208910

[49] Chen Y, Sun L, Ba X, Jiang S, Zhuang H, Zhu F, Wang H, Lan P, Shi Q, Wang Z, Chen Y, Shi K, Ji S, Jiang Y, Holmes MA, Yu Y. 2022. Epidemiology, evolution and cryptic susceptibility of methicillin-resistant Staphylococcus aureus in China: a whole-genome-based survey. Clin Microbiol Infect 28:85–92. doi:10.1016/j.cmi.2021.05.024 34022399

[50] Ersoy SC, Heithoff DM, Barnes L 5th, Tripp GK, House JK, Marth JD, Smith JW, Mahan MJ. 2017. Correcting a fundamental flaw in the paradigm for antimicrobial susceptibility testing. EBioMedicine 20:173–181. doi:10.1016/j.ebiom.2017.05.026 28579300 PMC5478264

[51] Petraitis V, Petraitiene R, Kavaliauskas P, Naing E, Garcia A, Sutherland C, Kau AY, Goldner N, Bulow C, Nicolau DP, Walsh TJ. 2021. Pharmacokinetics, tissue distribution, and efficacy of VIO-001 (meropenem/piperacillin/tazobactam) for treatment of methicillin-resistant Staphylococcus aureus bacteremia in immunocompetent rabbits with chronic indwelling vascular catheters. Antimicrob Agents Chemother 65:e0116821. doi:10.1128/AAC.01168-21 34460301 PMC8522724

[52] Ersoy SC, Abdelhady W, Li L, Chambers HF, Xiong YQ, Bayer AS. 2019. Bicarbonate resensitization of methicillin-resistant Staphylococcus aureus to β-lactam antibiotics. Antimicrob Agents Chemother 63:e00496-19. doi:10.1128/AAC.00496-19 31010857 PMC6591647

[53] Sollier J, Basler M, Broz P, Dittrich PS, Drescher K, Egli A, Harms A, Hierlemann A, Hiller S, King CG, et al.. 2024. Revitalizing antibiotic discovery and development through in vitro modelling of in-patient conditions. Nat Microbiol 9:1–3. doi:10.1038/s41564-023-01566-w 38177300

[54] Sakoulas G, Nizet V. 2024. Measuring beta-lactam minimum inhibitory concentrations in Staphylococcus aureus in the clinical microbiology laboratory: pinning the tail on the donkey. J Clin Microbiol 62:e0036623. doi:10.1128/jcm.00366-23 37966224 PMC10793257

[55] Sakoulas G, Geriak M, Nizet V. 2019. Is a reported penicillin allergy sufficient grounds to forgo the multidimensional antimicrobial benefits of β-lactam antibiotics? Clin Infect Dis 68:157–164. doi:10.1093/cid/ciy557 29986019 PMC6293005

[56] Sakoulas G, Okumura CY, Thienphrapa W, Olson J, Nonejuie P, Dam Q, Dhand A, Pogliano J, Yeaman MR, Hensler ME, Bayer AS, Nizet V. 2014. Nafcillin enhances innate immune-mediated killing of methicillin-resistant Staphylococcus aureus. J Mol Med (Berl) 92:139–149. doi:10.1007/s00109-013-1100-7 24297496 PMC3926703

[57] Davis JS, Hal S, Tong SY. 2015. Combination antibiotic treatment of serious methicillin-resistant Staphylococcus aureus infections. Semin Respir Crit Care Med 36:003–016. doi:10.1055/s-0034-1396906 25643267

[58] Yang SJ, Xiong YQ, Boyle-Vavra S, Daum R, Jones T, Bayer AS. 2010. Daptomycin-oxacillin combinations in treatment of experimental endocarditis caused by daptomycin-nonsusceptible strains of methicillin-resistant Staphylococcus aureus with evolving oxacillin susceptibility (the “seesaw effect”). Antimicrob Agents Chemother 54:3161–3169. doi:10.1128/AAC.00487-10 20547804 PMC2916313

[59] Sieradzki K, Tomasz A. 1997. Inhibition of cell wall turnover and autolysis by vancomycin in a highly vancomycin-resistant mutant of Staphylococcus aureus. J Bacteriol 179:2557–2566. doi:10.1128/jb.179.8.2557-2566.1997 9098053 PMC179004

[60] Tong SYC, Lye DC, Yahav D, Sud A, Robinson JO, Nelson J, Archuleta S, Roberts MA, Cass A, Paterson DL, et al.. 2020. Effect of vancomycin or daptomycin with vs without an antistaphylococcal β-lactam on mortality, bacteremia, relapse, or treatment failure in patients with MRSA bacteremia. A randomized clinical trial. JAMA 323:527–537. doi:10.1001/jama.2020.0103 32044943 PMC7042887

[61] Legg A, Meagher N, Johnson SA, Roberts MA, Cass A, Scheetz MH, Davies J, Roberts JA, Davis JS, Tong SYC. 2023. Risk factors for nephrotoxicity in methicillin-resistant Staphylococcus aureus bacteraemia: a post hoc analysis of the CAMERA2 trial. Clin Drug Investig 43:23–33. doi:10.1007/s40261-022-01204-z PMC9834357 36217068

[62] Finch NA, Zasowski EJ, Murray KP, Mynatt RP, Zhao JJ, Yost R, Pogue JM, Rybak MJ. 2017. A quasi-experiment to study the impact of vancomycin area under the concentration-time curve-guided dosing on vancomycin-associated nephrotoxicity. Antimicrob Agents Chemother 61:e01293-17. doi:10.1128/AAC.01293-17 PMC5700348 28923869

[63] Alosaimy S, Rybak MJ, Sakoulas G. 2024. Understanding vancomycin nephrotoxicity augmented by β-lactams: a synthesis of endosymbiosis, proximal renal tubule mitochondrial metabolism, and β-lactam chemistry. Lancet Infect Dis 24:e179–e188. doi:10.1016/S1473-3099(23)00432-2 37883984

[64] Dilworth TJ, Casapao AM, Ibrahim OM, Jacobs DM, Bowers DR, Beyda ND, Mercier R-C. 2019. Adjuvant β-lactam therapy combined with vancomycin for methicillin-resistant Staphylococcus aureus bacteremia: does β-lactam class matter? Antimicrob Agents Chemother 63:e02211-18. doi:10.1128/AAC.02211-18 30617094 PMC6395910

[65] Geriak M, Haddad F, Rizvi K, Rose W, Kullar R, LaPlante K, Yu M, Vasina L, Ouellette K, Zervos M, Nizet V, Sakoulas G. 2019. Clinical data on daptomycin plus ceftaroline versus standard of care monotherapy in the treatment of methicillin-resistant Staphylococcus aureus bacteremia. Antimicrob Agents Chemother 63:e02483-18. doi:10.1128/AAC.02483-18 30858203 PMC6496065

[66] CLSI. 2022. CLSI supplement M100. InPerformance standards for antimicrobial susceptibility testing, 32nded. Clinical and Laboratory Standards Institute.

[67] Testing TECovAS. 2022. Media preparation for EUCAST disk diffusion testing and for determination of MIC values by the broth microdilution method.

[68] Andrade-Figueiredo M, Leal-Balbino TC. 2016. Clonal diversity and epidemiological characteristics of Staphylococcus aureus: high prevalence of oxacillin-susceptible mecA-positive Staphylococcus aureus (OS-MRSA) associated with clinical isolates in Brazil. BMC Microbiol 16:115. doi:10.1186/s12866-016-0733-4 27325108 PMC4915036

[69] Chung M, Kim CK, Conceição T, Aires-De-Sousa M, De Lencastre H, Tomasz A. 2016. Heterogeneous oxacillin-resistant phenotypes and production of PBP2A by oxacillin-susceptible/mecA-positive MRSA strains from Africa. J Antimicrob Chemother 71:2804–2809. doi:10.1093/jac/dkw209 27278899 PMC5031915

[70] Hososaka Y, Hanaki H, Endo H, Suzuki Y, Nakae T, Nagasawa Z, Otsuka Y, Sunakawa K. 2007. Characterization of oxacillin-susceptible mecA-positive Staphylococcus aureus: a new type of MRSA. J Infect Chemother 13:79–86. doi:10.1007/s10156-006-0502-7 17458674

[71] Quijada NM, Hernández M, Oniciuc EA, Eiros JM, Fernández-Natal I, Wagner M, Rodríguez-Lázaro D. 2019. Oxacillin-susceptible mecA-positive Staphylococcus aureus associated with processed food in Europe. Food Microbiol 82:107–110. doi:10.1016/j.fm.2019.01.021 31027762

[72] Saeed K, Ahmad N, Dryden M, Cortes N, Marsh P, Sitjar A, Wyllie S, Bourne S, Hemming J, Jeppesen C, Green S. 2014. Oxacillin-susceptible methicillin-resistant Staphylococcus aureus (OS-MRSA), a hidden resistant mechanism among clinically significant isolates in the Wessex region/UK. Infection 42:843–847. doi:10.1007/s15010-014-0641-1 24919530

[73] Song Y, Cui L, Lv Y, Li Y, Xue F. 2017. Characterisation of clinical isolates of oxacillin-susceptible mecA-positive Staphylococcus aureus in China from 2009 to 2014. J Glob Antimicrob Resist 11:1–3. doi:10.1016/j.jgar.2017.05.009 28729204

[74] Proulx MK, Palace SG, Gandra S, Torres B, Weir S, Stiles T, Ellison RT III, Goguen JD. 2016. Reversion from methicillin susceptibility to methicillin resistance in Staphylococcus aureus during treatment of bacteremia. J Infect Dis 213:1041–1048. doi:10.1093/infdis/jiv512 26503983 PMC4760414

[75] Conceição T, Coelho C, de Lencastre H, Aires-de-Sousa M. 2015. Frequent occurrence of oxacillin-susceptible mecA-positive Staphylococcus aureus (OS-MRSA) strains in two African countries. J Antimicrob Chemother 70:3200–3204. doi:10.1093/jac/dkv261 26318189

[76] Duarte FC, Danelli T, Tavares ER, Morguette AEB, Kerbauy G, Grion CMC, Yamauchi LM, Perugini MRE, Yamada-Ogatta SF. 2019. Fatal sepsis caused by mecA-positive oxacillin-susceptible Staphylococcus aureus: first report in a tertiary hospital of southern Brazil. J Infect Chemother 25:293–297. doi:10.1016/j.jiac.2018.09.010 30482697

[77] Liu R, Zhang J, Du X, Lv Y, Gao X, Wang Y, Wang J. 2021. Clonal diversity, low-level and heterogeneous oxacillin resistance of oxacillin sensitive MRSA. Infect Drug Resist 14:661–669. doi:10.2147/IDR.S288991 33642870 PMC7903957

[78] Pardo L, Giudice G, Mota MI, Gutiérrez C, Varela A, Algorta G, Seija V, Galiana A, Aguerrebere P, Klein M, Varela G. 2022. Phenotypic and genotypic characterization of oxacillin-susceptible and mecA positive Staphylococcus aureus strains isolated in Uruguay. Rev Argent Microbiol 54:293–298. doi:10.1016/j.ram.2022.05.004 35725665

[79] Sakoulas G, Gold HS, Venkataraman L, DeGirolami PC, Eliopoulos GM, Qian Q. 2001. Methicillin-resistant Staphylococcus aureus: comparison of susceptibility testing methods and analysis of mecA-positive susceptible strains. J Clin Microbiol 39:3946–3951. doi:10.1128/JCM.39.11.3946-3951.2001 11682512 PMC88469

[80] Shukla SK, Ramaswamy SV, Conradt J, Stemper ME, Reich R, Reed KD, Graviss EA. 2004. Novel polymorphisms in mec genes and a new mec complex type in methicillin-resistant Staphylococcus aureus isolates obtained in rural Wisconsin. Antimicrob Agents Chemother 48:3080–3085. doi:10.1128/AAC.48.8.3080-3085.2004 15273123 PMC478485

[81] Chen FJ, Wang CH, Chen CY, Hsu YC, Wang KT. 2014. Role of the mecA gene in oxacillin resistance in a Staphylococcus aureus clinical strain with a pvl-positive ST59 genetic background. Antimicrob Agents Chemother 58:1047–1054. doi:10.1128/AAC.02045-13 24277044 PMC3910894

[82] Zhuang H, Zhu F, Lan P, Ji S, Sun L, Chen Y, Wang Z, Jiang S, Zhang L, Zhu Y, Jiang Y, Chen Y, Yu Y. 2021. A random forest model based on core genome allelic profiles of MRSA for penicillin plus potassium clavulanate susceptibility prediction. Microb Genom 7:000610. doi:10.1099/mgen.0.000610 34554083 PMC8715440

[83] Ender M, McCallum N, Berger-Bächi B. 2008. Impact of mecA promoter mutations on mecA expression and β-lactam resistance levels. Int J Med Microbiol 298:607–617. doi:10.1016/j.ijmm.2008.01.015 18456552

[84] Ersoy SC, Manna AC, Proctor RA, Chambers HF, Harrison EM, Bayer AS, Cheung A. 2022. The NaHCO_3_-responsive phenotype in methicillin-resistant Staphylococcus aureus (MRSA) is influenced by mecA genotype. Antimicrob Agents Chemother 66:e0025222. doi:10.1128/aac.00252-22 35575577 PMC9211399

[85] Petersiel N, Giulieri S, Daniel DS, Fan S-H, Ersoy SC, Davis JS, Bayer AS, Howden BP, Tong SYC, CAMERA2 study group. 2024. Genomic investigation and clinical correlates of the in vitro β-lactam: NaHCO_3_ responsiveness phenotype among methicillin-resistant Staphylococcus aureus isolates from a randomized clinical trial. Antimicrob Agents Chemother 68:e0021824. doi:10.1128/aac.00218-24 38837393 PMC11232399

[86] Ersoy SC, Rose WE, Patel R, Proctor RA, Chambers HF, Harrison EM, Pak Y, Bayer AS. 2021. A combined phenotypic-genotypic predictive algorithm for in vitro detection of bicarbonate: β-lactam sensitization among methicillin-resistant Staphylococcus aureus (MRSA). Antibiotics (Basel) 10:1089. doi:10.3390/antibiotics10091089 34572671 PMC8469475

[87] Goering RV, Swartzendruber EA, Obradovich AE, Tickler IA, Tenover FC. 2019. Emergence of oxacillin resistance in stealth methicillin-resistant Staphylococcus aureus due to mecA sequence instability. Antimicrob Agents Chemother 63:e00558-19. doi:10.1128/AAC.00558-19 31109981 PMC6658785

[88] Liang B, Xiong Z, Liang Z, Zhang C, Cai H, Long Y, Gao F, Wang J, Deng Q, Zhong H, Xie Y, Huang L, Gong S, Zhou Z. 2022. Genomic basis of occurrence of cryptic resistance among oxacillin- and cefoxitin-susceptible mecA-positive Staphylococcus aureus. Microbiol Spectr 10:e0029122. doi:10.1128/spectrum.00291-22 35608351 PMC9241717

[89] Forbes BA, Bombicino K, Plata K, Cuirolo A, Webber D, Bender CL, Rosato AE. 2008. Unusual form of oxacillin resistance in methicillin-resistant Staphylococcus aureus clinical strains. Diagn Microbiol Infect Dis 61:387–395. doi:10.1016/j.diagmicrobio.2008.04.003 18508224

[90] Ikonomidis A, Michail G, Vasdeki A, Labrou M, Karavasilis V, Stathopoulos C, Maniatis AN, Pournaras S. 2008. In vitro and in vivo evaluations of oxacillin efficiency against mecA-positive oxacillin-susceptible Staphylococcus aureus. Antimicrob Agents Chemother 52:3905–3908. doi:10.1128/AAC.00653-08 18694946 PMC2573153

[91] Labrou M, Michail G, Ntokou E, Pittaras TE, Pournaras S, Tsakris A. 2012. Activity of oxacillin versus that of vancomycin against oxacillin-susceptible mecA-positive Staphylococcus aureus clinical isolates evaluated by population analyses, time-kill assays, and a murine thigh infection model. Antimicrob Agents Chemother 56:3388–3391. doi:10.1128/AAC.00103-12 22430957 PMC3370787

[92] Jones D, Elshaboury RH, Munson E, Dilworth TJ. 2018. A retrospective analysis of treatment and clinical outcomes among patients with methicillin-susceptible Staphylococcus aureus bloodstream isolates possessing detectable mecA by a commercial PCR assay compared to patients with methicillin-resistant Staphylococcus aureus bloodstream isolates. Antimicrob Agents Chemother 62:e01396-17. doi:10.1128/AAC.01396-17 29038267 PMC5740325

[93] Rammelkamp CH, Maxon T. 1942. Resistance of Staphylococcus aureus to the action of penicillin. Proc Soc Exp Biol Med 51:386–389. doi:10.3181/00379727-51-13986

[94] Ba X, Harrison EM, Lovering AL, Gleadall N, Zadoks R, Parkhill J, Peacock SJ, Holden MTG, Paterson GK, Holmes MA. 2015. Old drugs to treat resistant bugs: methicillin-resistant Staphylococcus aureus isolates with mecC are susceptible to a combination of penicillin and clavulanic acid. Antimicrob Agents Chemother 59:7396–7404. doi:10.1128/AAC.01469-15 26392513 PMC4649192

[95] Ba X, Raisen CL, Zhou ZC, Harrison EM, Peacock SJ, Holmes MA. 2022. Simultaneously screening for methicillin-resistant Staphylococcus aureus and its susceptibility to potentiated penicillins. J Med Microbiol 71. doi:10.1099/jmm.0.001562 35867942

[96] Milne LM, Crow MR, Emptage AG, Selkon JB. 1993. Effects of culture media on detection of methicillin resistance in Staphylococcus aureus and coagulase negative staphylococci by disc diffusion methods. J Clin Pathol 46:394–397. doi:10.1136/jcp.46.5.394 8320317 PMC501243

[97] Chambers HF, Kartalija M, Sande M. 1995. Ampicillin, sulbactam, and rifampin combination treatment of experimental methicillin-resistant Staphylococcus aureus endocarditis in rabbits. J Infect Dis 171:897–902. doi:10.1093/infdis/171.4.897 7706817

[98] Cantoni L, Wenger A, Glauser MP, Bille J. 1989. Comparative efficacy of amoxicillin-clavulanate, cloxacillin, and vancomycin against methicillin-sensitive and methicillin-resistant Staphylococcus aureus endocarditis in rats. J Infect Dis 159:989–993. doi:10.1093/infdis/159.5.989 2708846

[99] Franciolli M, Bille J, Glauser MR, Moreillon P. 1991. β-lactam resistance mechanisms of methicillin-resistant Staphylococcus aureus. J Infect Dis 163:514–522. doi:10.1093/infdis/163.3.514 1995724

[100] Hirano L, Bayer AS. 1991. Beta-Lactam-beta-lactamase-inhibitor combinations are active in experimental endocarditis caused by beta-lactamase-producing oxacillin-resistant staphylococci. Antimicrob Agents Chemother 35:685–690. doi:10.1128/AAC.35.4.685 2069374 PMC245079

[101] Chambers HF, Sachdeva M, Kennedy S. 1990. Binding affinity for penicillin-binding protein 2a correlates with in activity of β-lactan antibiotics against methicillin-resistant Staphylococcus aureus. J Infect Dis 162:705–710. doi:10.1093/infdis/162.3.705 2387996

[102] Rose WE, Bienvenida AM, Xiong YQ, Chambers HF, Bayer AS, Ersoy SC. 2020. Ability of bicarbonate supplementation to sensitize selected methicillin-resistant Staphylococcus aureus strains to β-lactam antibiotics in an ex vivo simulated endocardial vegetation model. Antimicrob Agents Chemother 64:e02072-19. doi:10.1128/AAC.02072-19 31844004 PMC7038310

[103] Ersoy SC, Otmishi M, Milan VT, Li L, Pak Y, Mediavilla J, Chen L, Kreiswirth B, Chambers HF, Proctor RA, Xiong YQ, Fowler VG, Bayer AS. 2020. Scope and predictive genetic/phenotypic signatures of bicarbonate (NaHCO_3_) responsiveness and β-lactam sensitization in methicillin-resistant Staphylococcus aureus. Antimicrob Agents Chemother 64:e02445-19. doi:10.1128/AAC.02445-19 32041719 PMC7179597

[104] Ersoy SC, Madrigal SL, Chen L, Mediavilla J, Kreiswirth B, Flores EA, Miller LG, Xiong YQ, Harrison EM, Blane B, Peacock SJ, Patel R, Chambers HF, Bayer AS, Proctor RA. 2025. Phenotypic and genotypic correlates of the sodium bicarbonate-responsive phenotype among methicillin-resistant Staphylococcus aureus isolates from skin and soft-tissue infections. Clin Microbiol Infect 31:588–593. doi:10.1016/j.cmi.2024.11.034 39626860 PMC11913587

[105] Ersoy SC, Hanson BM, Proctor RA, Arias CA, Tran TT, Chambers HF, Bayer AS. 2021. Impact of bicarbonate-β-lactam exposures on methicillin-resistant Staphylococcus aureus (MRSA) gene expression in bicarbonate-β-lactam-responsive vs. non-responsive strains. Genes (Basel) 12:1650. doi:10.3390/genes12111650 34828256 PMC8619011

[106] Ersoy SC, Chambers HF, Proctor RA, Rosato AE, Mishra NN, Xiong YQ, Bayer AS. 2023. Impact of bicarbonate on PBP2a production, maturation, and functionality in methicillin-resistant Staphylococcus aureus (MRSA). Antimicrob Agents Chemother 65:e02621-20. doi:10.1128/AAC.02621-20 33649115 PMC8092911

[107] Ersoy SC, Gonçalves B, Cavaco G, Manna AC, Sobral RG, Nast CC, Proctor RA, Chambers HF, Cheung A, Bayer AS. 2022. Influence of sodium bicarbonate on wall teichoic acid synthesis and β-lactam sensitization in NaHCO_3_-responsive and nonresponsive methicillin-resistant Staphylococcus aureus. Microbiol Spectr 10:e0342222. doi:10.1128/spectrum.03422-22 36377886 PMC9769754

[108] Parsons JB, Westgeest AC, Conlon BP, Fowler VG. 2023. Persistent methicillin-resistant Staphylococcus aureus bacteremia: host, pathogen, and treatment. Antibiotics (Basel) 12:455. doi:10.3390/antibiotics12030455 36978320 PMC10044482

[109] Giulieri SG, Guérillot R, Holmes NE, Baines SL, Hachani A, Hayes AS, Daniel DS, Seemann T, Davis JS, Van Hal S, Tong SYC, Stinear TP, Howden BP. 2023. A statistical genomics framework to trace bacterial genomic predictors of clinical outcomes in Staphylococcus aureus bacteremia. Cell Rep 42:113069. doi:10.1016/j.celrep.2023.113069 37703880

[110] Memmi G, Filipe SR, Pinho MG, Fu Z, Cheung A. 2008. Staphylococcus aureus PBP4 is essential for β-lactam resistance in community-acquired methicillin-resistant strains. Antimicrob Agents Chemother 52:3955–3966. doi:10.1128/AAC.00049-08 18725435 PMC2573147

[111] Gonzales PR, Pesesky MW, Bouley R, Ballard A, Biddy BA, Suckow MA, Wolter WR, Schroeder VA, Burnham C-AD, Mobashery S, Chang M, Dantas G. 2015. Synergistic, collaterally sensitive β-lactam combinations suppress resistance in MRSA. Nat Chem Biol 11:855–861. doi:10.1038/nchembio.1911 26368589 PMC4618095

[112] Banerjee R, Fernandez MG, Enthaler N, Graml C, Greenwood-Quaintance KE, Patel R. 2013. Combinations of cefoxitin plus other β-lactams are synergistic in vitro against community associated methicillin-resistant Staphylococcus aureus. Eur J Clin Microbiol Infect Dis 32:827–833. doi:10.1007/s10096-013-1817-9 23340864

[113] Quach DT, Sakoulas G, Nizet V, Pogliano J, Pogliano K. 2016. Bacterial cytological profiling (BCP) as a rapid and accurate antimicrobial susceptibility testing method for Staphylococcus aureus. EBioMedicine 4:95–103. doi:10.1016/j.ebiom.2016.01.020 26981574 PMC4776060

[114] Reichmann NT, Pinho MG. 2017. Role of SCCmec type in resistance to the synergistic activity of oxacillin and cefoxitin in MRSA. Sci Rep 7:6154. doi:10.1038/s41598-017-06329-2 28733674 PMC5522475

[115] Mainardi JL, Gutmann L, Acar JF, Goldstein FW. 1995. Synergistic effect of amoxicillin and cefotaxime against Enterococcus faecalis. Antimicrob Agents Chemother 39:1984–1987. doi:10.1128/AAC.39.9.1984 8540703 PMC162868

[116] Sumita Y, Mitsuhashi S. 1991. In vitro synergistic activity between meropenem and other beta-lactams against methicillin-resistant Staphylococcus aureus. Eur J Clin Microbiol Infect Dis 10:77–84. doi:10.1007/BF01964412 1864279

[117] Yoneda A, Thänert R, Burnham C-A, Dantas G. 2020. In vitro activity of meropenem/piperacillin/tazobactam triple combination therapy against clinical isolates of Staphylococcus aureus, Staphylococcus epidermidis, Staphylococcus pseudintermedius and vancomycin-resistant Enterococcus spp. Int J Antimicrob Agents 55:105864. doi:10.1016/j.ijantimicag.2019.105864 31870598

[118] Gilbertie J, Ulloa ER, Daiker JC, Nguyen K, Smelter D, Rose W, Geriak M, Schnabel LV, Nizet V, Sakoulas G. 2022. Potent activity of ertapenem plus cefazolin within staphylococcal biofilms: a contributing factor in the treatment of methicillin-susceptible Staphylococcus aureus endocarditis. Open Forum Infect Dis 9:ofac159. doi:10.1093/ofid/ofac159 35493130 PMC9045957

[119] Ulloa ER, Singh KV, Geriak M, Haddad F, Murray BE, Nizet V, Sakoulas G. 2020. Cefazolin and ertapenem salvage therapy rapidly clears persistent methicillin-susceptible Staphylococcus aureus bacteremia. Clin Infect Dis 71:1413–1418. doi:10.1093/cid/ciz995 31773134 PMC7486850

